# Healthy Lifestyle Behaviours Are Associated with Children’s Psychological Health: A Cross-Sectional Study

**DOI:** 10.3390/ijerph17207509

**Published:** 2020-10-15

**Authors:** Margaret M. Thomas, Jessica Gugusheff, Heather J. Baldwin, Joanne Gale, Sinead Boylan, Seema Mihrshahi

**Affiliations:** 1 Prevention Research Collaboration, Sydney School of Public Health, Faculty of Medicine and Health, The University of Sydney, Sydney 2006, Australia; jessica.gugusheff@health.nsw.gov.au (J.G.); heather.baldwin@sydney.edu.au (H.J.B.); joanne.gale@sydney.edu.au (J.G.); sinead.boylan@sydney.edu.au (S.B.); 2 Centre for Epidemiology and Evidence, NSW Ministry of Health, St Leonards 2065, Australia; 3 Department of Health Systems and Populations, Faculty of Medicine, Health and Human Sciences, Macquarie University, North Ryde 2109, Australia; seema.mihrshahi@mq.edu.au

**Keywords:** children, adolescents, diet, physical activity, screen time, mental health, psychological factors

## Abstract

Protecting children’s mental health is important and studies have shown that diet and exercise can have a positive impact. There are limited data available, however, from representative populations of children on the relationship between regular healthy lifestyle behaviours and psychological health. Data were obtained from the New South Wales Child Population Health Survey, 2013–2014. Parents were asked about diet, physical activity and screen time behaviours and completed the Strengths and Difficulties Questionnaire (SDQ) for one child aged 5–15. Higher SDQ scores indicate poorer psychological health and risk for mental health problems. Multivariable linear and logistic regression examined the relationships among dietary consumption, physical activity, screen time and SDQ scores, adjusting for potential confounding. Meeting screen time recommendations was most strongly associated with a lower SDQ total difficulties score (5–10 years: −1.56 (−2.68, −0.44); 11–15 years: −2.12 (−3.11, −1.12)). Children and adolescents who met screen time recommendations were also significantly less likely to have any score in the at-risk range. Children and adolescents meeting vegetable intake guidelines had significantly lower total difficulties scores (5–10 years: −1.54 (−3.03, −0.05); 11–15 years: −1.19 (−3.60, −0.39)), as did adolescents meeting discretionary food guidelines (−1.16 (−2.14, −0.18)) and children consuming the recommended fruit intake (−1.26 (−2.42, −0.10)). Our findings indicate that more effective interventions to increase the proportion of young Australians who meet the guidelines for diet and screen time would contribute to protecting their mental health.

## 1. Introduction

There are concerns about the prevalence of mental health problems among Australia’s young people, with a large-scale study identifying mild to severe mental health disorders in 14% of 4–17 years old [1]. International reviews have found that healthy eating and physical activity in children and adolescents are associated with better mental health [2,3,4,5]. In addition, unhealthy diets, inadequate amounts of physical activity and increased sedentary behaviour in children contribute to the development of obesity and other cardiovascular risk factors [6]. Investigations into unhealthy lifestyle behaviours have shown that they contribute to poorer health related quality of life and have a prolonged negative influence on psychological health [2,7,8,9]. Increased electronic screen time is an issue of growing concern across all developed countries [10,11], due to evidence of its contribution to increased sedentary behaviour and a host of other undesirable outcomes, including poorer quality of life and behavioural and psychological difficulties [12,13,14,15]. 

Among younger Australian children, a higher quality diet has been associated with better mental health [16], whilst greater physical activity has been linked to fewer depressive symptoms in adolescents [17]. High levels of discretionary food intake [18,19] and reduced consumption of fresh fruit and leafy green vegetables [20] have both been linked with adverse adolescent mental health outcomes. Studies indicate that there is a high prevalence of some unhealthy lifestyle behaviours among Australian young people, for example, 45–80% of 8–16 years old exceed the recommended screen time, around half have excess discretionary food intake and around 90% or more do not meet vegetable consumption guidelines, indicating that many Australian children may be at risk of experiencing sub-optimal mental health [11,21,22]. Differences in lifestyle behaviours between children and adolescents supports the need to examine mental health outcomes in these age groups separately [21]. Our research focusses on the actual reported lifestyle behaviour, and reported psychological health of a representative sample of Australian children living in the community, in order to determine which healthy behaviours have the most impact and also to examine the differences between children and adolescents. 

Childhood and adolescence is likely to be a critical time for establishing good mental health but there is still much to be learned about factors that may have a positive impact on psychological health in childhood [23,24]. There are still inconsistencies and gaps in the evidence for the relationship between children’s health behaviours and mental health, thus more research is needed [5]. While some studies have pointed to the value of an overall healthy lifestyle [8], there is currently an inadequate understanding of the relative importance of the different behaviours that make up a healthy lifestyle, and how each contributes to children’s psychological health. Additionally, research has frequently been conducted among select samples, and often without adequate control for confounding factors [4], so more sound analysis of population-based data is needed. The aim of this analysis was to examine the association between key healthy lifestyle behaviours—diet, physical activity and recreational screen time—and risk for mental health problems in a representative population of children aged 5–15 living in New South Wales, Australia.

## 2. Materials and Methods 

### 2.1. Data Source

Data were obtained from the New South Wales (NSW) Child Population Health Survey (CPHS), 2013–2014 [25]. The CPHS is a component of the NSW Population Health Survey (PHS), an ongoing survey of health and health related behaviour among people living in NSW, Australia’s most populous state, comprising approximately one third of the Australian population. The NSW Ministry of Health carries out the survey between February and December each year using computer-assisted telephone interviewing and randomly selected landline and mobile phone numbers of households in the community. The data are weighted in order to be representative of the NSW population and to account for the probability of selection.

The CPHS is completed by a parent on behalf of one child or adolescent in their care, aged between 0 and 15, with the Strengths and Difficulties Questionnaire (SDQ) component completed by parents of children aged 4–15. The SDQ was included in the CPHS in consecutive years 2013–2014 but has not been included since that time, therefore these were the most recent data available. Survey participants provide verbal informed consent and the survey has been approved by the NSW Population and Health Services Research Ethics Committee. Further information on the survey and methods are available from the NSW Ministry of Health Child Population Survey website [25]. Our study was restricted to children aged 5–15 years since parents were only asked about physical activity for children 5–15 years. No other inclusion or exclusion criteria were applied for the secondary analysis undertaken.

Ethical approval for the NSW Child Population Health Survey was provided by the NSW Population Health Services and Research Ethics Committee (AU RED Reference: HREC/11/CIPHS/55. Cancer Institute NSW reference number: 2011/09/349). A parent or caregiver provided informed consent and answered the survey questions for a randomly selected child. This study was conducted in accordance with the Declaration of Helsinki.

### 2.2. Exposure Measures

*Diet:* For dietary measures, parents were asked to estimate the number of serves of various types of foods and beverages their child usually consumed per day. A serve of fruit was defined as one medium or two small pieces of fruit, or one cup of diced pieces, and a serve of vegetables was defined as half a cup of cooked or one cup of salad vegetables. Intake of discretionary foods [26] was estimated based on questions about the number of times in a day/week/month children ate the particular foods. Discretionary foods included processed meat, salty snacks, fried potato products, sugary baked goods, fast food, cordial or other sugar-sweetened beverages and confectionary [26]. For each discretionary food, where respondents provided a weekly or monthly report of number of times the food was consumed, these were divided by 7 and 30, respectively, to provide an estimated daily number of serves. To estimate the total amount of serves of discretionary food per day, serves per day for all the discretionary foods were summed. Appropriate discretionary food consumption was defined as consuming these foods less than three times per week, following Boylan et al. [21]. Dietary indicators for fruit and vegetable consumption were based on meeting age-appropriate Australian Dietary Guidelines (Table S1) [27]. As survey answers were provided in whole numbers, half serves in the recommendations were rounded up to whole serves. This only applied to girls and boys aged 5–8 for fruit and vegetable consumption and boys aged 12–15 for vegetable consumption (see Table S1). 

*Activity:* For activity measures, parents were asked to estimate the amount of time their child spent doing physical activity during school hours, outside of school hours and on the weekend, from which total daily average estimates for time spent in physical activity were calculated. Similarly, parents estimated the amount of time their child engaged in screen time activities at home (TV, videos/DVDs and computer games) during the school week and on weekends and total daily average estimates for screen time were calculated. Physical activity and screen time indicators were based on meeting Australia’s Physical Activity and Sedentary Behaviour Guidelines for children and adolescents (see Table S1) [28,29]. 

### 2.3. Outcome Measures

The outcome measures were based on the SDQ which was part of the CPHS questionnaire in 2013–2014 and completed by all consenting parents caring for a child aged 4–15. The SDQ is a behavioural screening tool for assessing child and adolescent mental health [30] and has been used successfully in populations of Australian children [16,31]. A higher total score relates to a higher level of psychological distress (maximum score of 40) and is predictive of diagnosed mental health problems [30]. The SDQ comprises 25 attributes on five scales: (1) emotional symptoms; (2) conduct problems; (3) hyperactivity or inattention; (4) peer relationship problems; and (5) prosocial behaviour. A total difficulties score is obtained by adding scores from the first four scales. A score of 5–10 for emotional symptoms, 7–10 for hyperactivity/inattention, 4–10 for conduct or peer relationship problems and 0–4 for prosocial behaviour indicates a child is at risk of experiencing problems in that area. 

### 2.4. Confounders

Potential confounders included in the analysis were age, sex, body mass index (BMI), mother’s highest education qualification (indicator of socioeconomic status), family structure (single parent family) and remoteness of residence. Residential postcode was used to determine remoteness through the Accessibility/Remoteness Index of Australia (ARIA) [32].

### 2.5. Statistical Methods

Multivariable logistic regression models were used to test whether meeting recommendations for each of these lifestyle behaviours was associated with having a score in the at-risk range in any of the five SDQ sub-scales. Logistic regression was also used to examine the association between each diet or activity behaviour and having an at-risk score in each sub-scale individually. Adjustments for demographic confounders were included in each model. A multivariable linear regression model was used to test for an association between the diet and activity behaviours and the total difficulties score. The model included all five diet and activity recommendations, to control for the effect of each recommendation on the other, as well as demographic confounders. Regression models for problems in individual SDQ domains were not analysed due to low cell numbers. Survey weights were included in all models, with models run using the Survey Package in R [33]. Children/adolescents missing data for any variable included in a model were excluded from that model.

As the distribution of the total difficulties score was right-skewed, a sensitivity analysis was performed using a square-root transformation. The sensitivity analysis confirmed only negligible differences between the transformed and raw scores, so analysis using raw scores is presented. 

## 3. Results

### 3.1. Sample Characteristics

Of 2665 respondents (2013: *n* = 1247; 2014: *n* = 1418), 2644 (99.2%) had complete age, sex and SDQ data. Among the children (aged 5–10 years), 1341 (98.1%) had complete diet information, and 1277 (93.4%) had complete activity information, while, among adolescents (aged 11–15 years), 1257 (98.4%) had complete diet information and 1183 (92.6%) had complete activity information. Characteristics of the survey participants are summarised in Table 1. Males made up 50.0% of child and 51.1% of adolescent respondents. Most children met the fruit recommendations compared to just over half the adolescents, whilst only very small proportions across both age groups met vegetable intake recommendations. Physical activity recommendations, to be active for 60 min or more each day, were met by 74.9% children and 66.9% of adolescents. Lower proportions of both children (65.6%) and adolescents (51.6%) met screen time recommendations. 

Population level mean and median fruit, vegetable and discretionary food intake, along with hours per day of screen time and physical activity, are reported in Table 2. Both children (aged 5–10 years) and adolescents (aged 11–15 years) consumed an average of two serves of fruit and two serves of vegetables per day. The median screen time for children per day was 1.5 h (IQR = 1.0–2.3), whilst adolescents spent approximately two hours a day in front of a screen (mean = 2.1, SE = 0.06, median = 1.8, IQR (1.1–2.5). 

### 3.2. SDQ Sub-Scales

In the crude analysis, children (aged 5–10) who met the discretionary food recommendations and both children and adolescents (aged 11–15) who met the screen time recommendations were significantly less likely to have any SDQ score in the at-risk range (Table 3). After adjustment for potential confounders, the significantly lower likelihood of any at-risk score on the SDQ remained only for meeting the screen time recommendations, in both children and adolescents (Table 3). 

Furthermore, meeting screen time recommendations was significantly associated with a reduced likelihood of an at-risk score in each of the SDQ sub-scales for children and two of the five subscales for adolescents (Table S2).

### 3.3. SDQ Total Difficulties Score

The mean total difficulties score on the SDQ was 7.8 (SE = 0.25, median = 6.7, IQR = 3.8–10.4) for children (aged 5–10), and 7.4 (SE = 0.25, median = 5.8, IQR = 2.9–9.8) for adolescents (aged 11–15). Meeting the vegetable intake recommendations or screen time recommendations was significantly associated with a lower total difficulties score in both age groups after adjustment for potential confounders (Table 4).

Meeting fruit recommendations in children and discretionary food intake in adolescents was also associated with a significantly reduced total difficulties score. A multivariable analysis including all diet and activity indicators in a single model, adjusted for demographic factors, showed that after accounting for other health behaviours, only meeting screen time recommendations had a significant association with a lower total difficulties score in both age groups (Table 5). Meeting the vegetable intake recommendation was also significantly associated with a lower total difficulties score after adjusting for the other indicators, for adolescents only (Table 5). Model estimates for demographic confounders are provided in Table S3.

## 4. Discussion

Our analysis is the first Australian study of a large representative population-based sample of children and adolescents showing that those who have healthy lifestyle behaviours, especially appropriate screen time and healthier diets, are less likely to experience psychological difficulties. For children and adolescents who met recommendations for screen time, there was a significant and consistent benefit in terms of a reduced SDQ total difficulties score, indicating better mental health. The results for diet were slightly different for adolescents and children and showed some variation in the effects of the different dietary behaviours, but indicate a benefit for meeting at least some dietary recommendations. Since even a small increase in the SDQ total difficulties score has been shown to indicate increased risk of diagnosed mental health problems [30], maintaining a healthy lifestyle may prevent the development of psychological difficulties or mental health problems in children and adolescents. Within the context of concerns about mental health problems among children, these findings indicate the importance of implementing policy and program interventions to improve children’s diets and reduce the time they spend using electronic screens. 

We demonstrated that, of all the included healthy lifestyle behaviours, screen time above recommended levels was most strongly associated with a higher total difficulties score and therefore risk for poorer mental health outcomes. This is in line with other studies which have shown a relationship between greater screen time and poorer quality of life, depression and other mental health problems [10,34,35,36]. Amounts of screen time and mental health are related [3,37], most probably bi-directionally: poorer initial mental health predicts higher screen time (and declining physical activity levels), whilst increases in anxiety are associated with increasing screen time [9,38,39]. Compared to children, we identified that proportionally fewer adolescents met the screen time recommendations, in agreement other Australian population studies [22] and international findings [10]. This reinforces adolescence as a particularly critical time for reducing mental health related risk behaviours. 

We did not find a statistically significant association between physical activity and mental health, but we did observe a tendency for both children and adolescents who met physical activity recommendations to have lower SDQ scores. Some researchers have found evidence for an association between physical activity and lower rates of depression and anxiety, or more positive self-perceptions and self-esteem, but a review concluded that the evidence base is limited [3,24]. It may also be important to differentiate between types of physical activity since many studies showing a positive relationship used acute exercise sessions as the exposure variable [40], whereas we examined regular physical activity participation that met recommended levels. A similar need to examine activity types, intensity and contexts has been found in adult studies [41].

Adequate fruit and vegetable intake and lower discretionary food consumption were associated with a lower total difficulties score, although somewhat differentially among children and adolescents. Low discretionary food intake appears to be more important for the psychological health of adolescents, and this is consistent with previous studies, which have also shown that discretionary foods play a significant role in adolescent mental health [19,20,42]. Several studies have found an association between an unhealthy diet and mental health in children and/or adolescents, and, while measures differed, high consumption of discretionary foods has often been used to define an unhealthy diet [4]. For children in our study, eating sufficient amounts of fruit was important for mental health, which has also been shown in other studies [2]. 

Few studies have examined in detail the contribution of fruit and vegetable consumption to good mental health [4]. Oddy et al. noted a positive relationship for fruit and leafy green vegetables but not for other types of vegetables [20], while Renzaho et al. found no relationship between vegetable consumption and SDQ scores, although they did not examine the effect of meeting guidelines for fruit and vegetables [43]. Nevertheless, only a very small proportion of NSW children and adolescents are meeting the guidelines for vegetable consumption, consistent with our previous research in the Australian National Health Surveys [44], so our findings indicate that few children and adolescents are receiving the potential mental health benefits. 

Over half of the children and adolescents met the screen time recommendations. This is based on parent self-report, and it is likely that the amount of screen time use among children and adolescents in NSW is much higher than identified in the CPHS due to poor parent awareness or bias in reporting in the CPHS [22]. Since our study included a number of healthy lifestyle behaviours, we were able to identify that large amounts of screen time have a larger negative impact on psychological health than diet or physical activity behaviours. Efforts should therefore be made to improve estimations of screen time use, particularly as the situation is likely to have worsened in recent years because of the proliferation of screen devices now used by children and adolescents. This study provides evidence of a relationship between meeting recommendations for healthy lifestyle behaviours and having better results on the SDQ for NSW children aged 5–15 years, and it adds to the findings of previous studies about the importance of these lifestyle behaviours for mental as well as physical health in children and young people [2,7,16]. 

A key strength of our study is that we used the results of a validated child psychological difficulties questionnaire, the SDQ, in a population sample of children and adolescents to examine if there were associations with important regular healthy lifestyle behaviours. Our analysis adjusted for a wide range of socioeconomic and family factors (such as parent’s education and single parent families) which are independently associated with diet, physical activity and overall healthy lifestyle. We also adjusted for BMI, as overweight and obesity is likely to be an important confounder in the diet and mental health relationship [45]. These adjustments increase the reliability of our findings. We used the linear scale for the SDQ total difficulties score, rather than cut-offs for diagnosed mental health problems [30], since our aim was to examine whether differences in scores were associated with the healthy lifestyle behaviours. The SDQ is an appropriate tool for measuring psychological difficulties in children as it also captures issues less serious than diagnosed mental health problems, but the results can predict risk for the development of more serious problems. [30]. The mean total difficulties score for our sample of NSW children was similar to the mean found for parent report in an earlier Australian study [31]. Our study used a large representative population-based sample of Australian children in the most populated state of Australia which provides some confidence that the results may be generalisable to other children of the same age. 

Our analysis, however, has some limitations. The proportions meeting the healthy food guidelines may have been slightly underestimated since the survey questions were unable to detect half serves and therefore the recommendations were increased to be a whole serve for a portion of the sample but this is very unlikely to have impacted the findings. In the dietary guidelines for children, a serve of discretionary food is based on kilojoules, so the amount is different for each type of food and we were unable to take this into account. This may have resulted in underestimating the proportion of children meeting discretionary food guidelines. We were also not able to adjust for overall energy intake as this data was not collected. 

Data were based on parent report, which may have over or underestimated diet and activity according to social desirability bias or lack of information (e.g., difficulty estimating physical activity during school hours). Self-reported data on screen time in adolescents show a lower prevalence of meeting guidelines [22] compared to parent report, and psychological difficulties may be more likely to be reported by children themselves compared to parent report [31], suggesting that unhealthy behaviours and difficulties may have both been underestimated. 

In addition, there was a higher proportion of missing data for the screen time indicator (7%) compared to other variables and the questions used in 2013/2014 may not have adequately captured the full range of screen time activities, particularly leisure screen time on hand held devices, although this was likely far less common in 2013/2014 than now. Our data are cross-sectional and therefore reverse causality, or a bi-directional effect, cannot be ruled out. Although our data captured information from over 2600 children, they were collected in one state of Australia and analysis of similar large, population-based samples of children in other states would provide additional valuable evidence for confirming the results of our study. 

## 5. Conclusions

Our research indicates that NSW children and adolescents who have better dietary behaviours and lower screen time have better psychological health. The strong association between appropriate screen time and better psychological health observed in this study is particularly important in view of rising concerns about the impact of screen time, particularly small screens, on children’s mental health and well-being [10,46]. The results strengthen the evidence for the association between lifestyle behaviours and child mental health and highlight the importance of implementing policies and interventions to support children and families to achieve healthy lifestyles.

## Figures and Tables

**Table 1:** Survey sample characteristics, food and activity behaviours and emotional and behavioural problems from the NSW Child Population Health Survey, 2013–2014.

Characteristic	5–10 Years *N* = 1367		11–15 Years *N* = 1277	
	*n*	%	*n*	%
**Sex**				
Male	684	50.0	653	51.1
Female	683	50.0	624	48.9
**Mother’s highest qualification** ^§^				
Year 10 certificate or diploma	143	11.2	195	16.3
Completed High School (HSC)	158	12.3	141	11.8
Tertiary certificate or diploma	363	28.3	383	32.1
University or other tertiary degree	619	48.3	475	39.8
**Body Mass Index**				
Overweight or obese	220	24.2	213	20.8
Single parent family	318	23.3	356	27.9
**Remoteness**				
Major cities	769	56.3	702	54.9
Regional or remote	598	43.7	576	45.1
**Food and activity behaviours**				
Met fruit recommendation	971	71.7	736	58.1
Met vegetable recommendation	80	5.9	60	4.8
Met discretionary food cut off	716	52.4	626	49.0
Met PA recommendation	1016	74.9	849	66.9
Met screen time recommendation	840	65.6	612	51.6
**Emotional and behavioural problems**				
Emotional symptoms	144	10.5	198	15.5
Conduct problems	105	7.7	85	6.7
Hyperactivity-inattention	163	11.9	124	9.7
Peer problems	105	7.7	167	13.1
Prosocial behaviour	28	2.1	37	2.9
Any problems ^β^	333	24.3	338	26.5

Note: **^§^** 167 children (6.3%) missing data; ^β^ At least one problem score in any of the SDQ scales; there were higher levels of missing data for screen time (87 (6.3%) for 5–10 years old and 91 (7.1%) for 11–15 years old).

**Table 2:** Mean and median values for dietary and activity behaviours stratified by age.

Behaviour	5–10 Years		11–15 Years	
	Mean (SE)	Median (IQR)	Mean (SE)	Median (IQR)
Fruit serves per day	2.1 (0.04)	1.9 (1.0–2.6)	1.9 (0.07)	1.8 (0.9–2.0)
Vegetable serves per day	2.0 (0.05)	1.8 (0.9–2.9)	2.2 (0.06)	1.8 (0.9–2.9)
Discretionary food serves per day	0.6 (0.03)	0.0 (0.0–0.6)	0.7 (0.03)	0.0 (0.0–0.7)
PA hours per day	1.9 (0.06)	1.5 (0.9–2.3)	1.6 (0.06)	1.3 (0.7–2.1)
Screen time hours per day	1.8 (0.05)	1.5 (1.0–2.3)	2.1 (0.06)	1.8 (1.1–2.5)

**Table 3:** Unadjusted and adjusted odds ratios of having any emotional, behavioural or social problem^#^ as assessed by the Strengths and Difficulties Questionnaire (SDQ), by diet and physical activity indicators.

Indicator	Any Problem		Any Problem	
	Unadjusted OR (95% CI)		Adjusted OR (95% CI)	
	5–10 Years	11–15 Years	5–10 Years	11–15 Years
Fruit serves per day	0.72 (0.49–1.05)	0.80 (0.55–1.18)	0.74 (0.45–1.24)	0.98 (0.62–1.54)
Vegetable serves per day	0.54 (0.23–1.25)	0.92 (0.41–2.07)	0.39 (0.13–1.16)	0.86 (0.31–2.35)
Discretionary food serves per day	0.60 (0.42–0.88) *	0.81 (0.55–1.19)	0.76 (0.47–1.25)	0.85 (0.51–1.25)
PA hours per day	0.84 (0.58–1.23)	0.69 (0.46–1.05)	0.90 (0.55–1.49)	0.68 (0.41–1.12)
Screen time hours per day	0.40 (0.27–0.59) *	0.54 (0.36–0.82) *	0.52 (0.32–0.87) *	0.48 (0.29–0.79) *

* *p* < 0.05, each indicator was included in a separate model, adjusted for age, sex, BMI, mother’s highest qualification, family structure (single parent family) and remoteness; ^#^ scored in the “at risk” range for at least one of the 25 attribute problems on the SDQ.

**Table 4:** Unadjusted and adjusted mean differences in total difficulties score, between those who met the diet and physical activity indicators.

Indicator	Unadjusted Mean Difference (95%CI)		Adjusted Mean Difference (95%CI)	
	5–10 Years	11–15 Years	5–10 Years	11–15 Years
Met fruit recommendations	−1.18 (−2.22, −0.15) *	−0.65 (−1.63,0.34)	−1.26 (−2.42, −0.10) *	0.00 (−1.06,1.05)
Met vegetable recommendations	−1.64 (−2.99, −0.30) *	−2.06 (−3.61, −0.51) *	−1.54 (−3.03, −0.05) *	−1.19 (−3.60, −0.39) *
Met discretionary food limit	−1.25 (−2.21, −0.29) *	−1.31 (−2.29, −0.34) *	−0.96 (−1.97,0.04)	−1.16 (−2.14, −0.18) *
Met PA recommendations	−0.90 (−1.91,0.12)	−0.86 (−1.86,0.15)	−0.74 (−1.76,0.28)	−0.68 (−1.62,0.26)
Met screen time recommendations	−2.69 (−3.81, −1.57) *	−2.23 (−3.24, −1.21) *	−1.60 (−2.70, −0.50) *	−2.18 (−1.36, −1.20) *

* *p* < 0.05, each indicator was included in a separate model, adjusted for year of age, sex, BMI, mother’s highest qualification, family structure (single parent family) and remoteness.

**Table 5:** Multivariable analysis of diet and physical activity factors predictive of total difficulties score in SDQ for children/adolescents aged 5–15 years, stratified by age.

Indicator	Mean Total Difficulties Score Difference (95%CI)	
	5–10 Years	11–15 Years
Met fruit recommendations	−0.90 (−2.10, 0.30)	0.26 (−0.86, 1.38)
Met vegetable recommendations	−0.90 (−2.47, 0.66)	−1.77 (−3.53, −0.01) *
Met discretionary food limit	−0.67 (−1.74, 0.39)	−0.95 (−1.97, 0.08)
Met PA recommendations	−0.30 (−1.34, 0.74)	−0.44 (−1.42, 0.55)
Met screen time recommendations	−1.56 (−2.68, −0.44) *	−2.12 (−3.11, −1.12) **

** *p* < 0.001, * *p* < 0.05. All factors were included in a single multivariable model, adjusted for age, sex, BMI, mother’s highest qualification family structure (single parent family) and remoteness. Estimates for demographic factors are provided in Table S3.

## Supplementary Materials

The following are available online at https://www.mdpi.com/1660-4601/17/20/7509/s1, Table S1: Healthy lifestyle behaviour cut-offs used in the analysis (half serves in the recommendations were rounded up to whole serves). Table S2: Unadjusted associations between diet/physical activity indicators and scoring in the at-risk range for each of the Strength and Difficulty Questionnaire (SDQ) subscales; Table S3: Demographics included in multivariable analysis of diet and physical activity factors predictive of total difficulties score in children aged 5–15 years, stratified by age.

## References

[1] Lawrence D., Johnson S., Hafekost J., Boterhoven de Haan K., Sawyer M., Ainley J., Zubrick S.R. (2015). The Mental Health of Children and Adolescents: Report on the Second Australian Child and Adolescent Survey of Mental Health and Wellbeing.

[2] O’Neil A., Quirk S.E., Housden S., Brennan S.L., Williams L.J., Pasco J.A., Berk M., Jacka F.N. (2014). Relationship Between Diet and Mental Health in Children and Adolescents: A Systematic Review. Am. J. Public Health.

[3] Biddle S.J., Asare M. (2011). Physical activity and mental health in children and adolescents: A review of reviews. Br. J. Sports Med..

[4] Khalid S., Williams C.M., Reynolds S.A. (2016). Is there an association between diet and depression in children and adolescents? A systematic review. Br. J. Nutr..

[5] Biddle S.J., Ciaccioni S., Thomas G., Vergeer I. (2019). Physical activity and mental health in children and adolescents: An updated review of reviews and an analysis of causality. Psychol. Sport Exerc..

[6] Mihrshahi S., Gow M.L., Baur L.A. (2018). Contemporary approaches to the prevention and management of paediatric obesity: An Australian focus. Med. J. Aust..

[7] Wu X., Zhuang L., Li W., Guo H., Zhang J., Zhao Y., Hu J., Gao Q., Luo S., Ohinmaa A. (2019). The influence of diet quality and dietary behavior on health-related quality of life in the general population of children and adolescents: A systematic review and meta-analysis. Qual. Life Res..

[8] Dumuid D., Olds T., Lewis L.K., Martin-Fernández J.A., Katzmarzyk P.T., Barreira T., Broyles S.T., Chaput J.-P., Fogelholm M., Hu G. (2017). Health-related quality of life and lifestyle behavior clusters in school-aged children from 12 countries. J. Pediatr..

[9] Herman K.M., Hopman W.M., Sabiston C.M. (2015). Physical activity, screen time and self-rated health and mental health in Canadian adolescents. Prev. Med..

[10] Saunders T.J., Vallance J.K. (2017). Screen time and health indicators among children and youth: Current evidence, limitations and future directions. Appl. Health Econ. Health Policy.

[11] Houghton S., Hunter S.C., Rosenberg M., Wood L., Zadow C., Martin K., Shilton T. (2015). Virtually impossible: Limiting Australian children and adolescents daily screen based media use. BMC Public Health.

[12] Ghasemi S.R., Rajabi Gilan N., Reshadat S., Hemati A. (2019). Investigating Health-Related Quality of Life and the Use of Media Technologies in Adolescents. J. Maz. Univ. Med. Sci..

[13] Suchert V., Hanewinkel R., Isensee B. (2015). Sedentary behavior and indicators of mental health in school-aged children and adolescents: A systematic review. Prev. Med..

[14] Hoare E., Milton K., Foster C., Allender S. (2016). The associations between sedentary behaviour and mental health among adolescents: A systematic review. Int. J. Behav. Nutr. Phys. Act..

[15] Sigman A. (2012). Time for a view on screen time. Arch. Dis. Child..

[16] Dimov S., Mundy L.K., Bayer J.K., Jacka F.N., Canterford L., Patton G.C. (2019). Diet quality and mental health problems in late childhood. Nutr. Neurosci..

[17] Kremer P., Elshaug C., Leslie E., Toumbourou J.W., Patton G.C., Williams J. (2014). Physical activity, leisure-time screen use and depression among children and young adolescents. J. Sci. Med. Sport.

[18] Jacka F.N., Kremer P.J., Leslie E.R., Berk M., Patton G.C., Toumbourou J.W., Williams J.W. (2010). Associations between diet quality and depressed mood in adolescents: Results from the Australian Healthy Neighbourhoods Study. Aust. N. Z. J. Psychiatry.

[19] Jacka F.N., Kremer P.J., Berk M., de Silva-Sanigorski A.M., Moodie M., Leslie E.R., Pasco J.A., Swinburn B.A. (2011). A prospective study of diet quality and mental health in adolescents. PLoS ONE.

[20] Oddy W.H., Robinson M., Ambrosini G.L., Therese A., de Klerk N.H., Beilin L.J., Silburn S.R., Zubrick S.R., Stanley F.J. (2009). The association between dietary patterns and mental health in early adolescence. Prev. Med..

[21] Boylan S., Hardy L., Drayton B., Grunseit A., Mihrshahi S. (2017). Assessing junk food consumption among Australian children: Trends and associated characteristics from a cross-sectional study. BMC Public Health.

[22] Hardy L., Mihrshahi S., Drayton B., Bauman A. (2016). NSW Schools Physical Activity and Nutrition Survey (SPANS): Full Report.

[23] Sawyer S.M., Afifi R.A., Bearinger L.H., Blakemore S.-J., Dick B., Ezeh A.C., Patton G.C. (2012). Adolescence: A foundation for future health. Lancet.

[24] Lubans D., Richards J., Hillman C., Faulkner G., Beauchamp M., Nilsson M., Kelly P., Smith J., Raine L., Biddle S. (2016). Physical activity for cognitive and mental health in youth: A systematic review of mechanisms. Pediatrics.

[25] NSW Health Child Population Health Survey. https://www.health.nsw.gov.au/surveys/child/Pages/default.aspx.

[26] National Health and Medical Research Council (Australia) (2013). Australian Dietary Guidelines.

[27] National Health and Medical Research Council (Australia) Australian Dietary Guidelines (updated July 2019). https://www.nhmrc.gov.au/_files_nhmrc/file/your_health/healthy/nutrition/n55a_australian_dietary_guidelines_summary_131014_1.pdf.

[28] Department of Health (2018). Australia’s Physical Activity and Sedentary Behaviour Guidelines. Fact Sheet: Children (5–12 Years).

[29] Department of Health (2018). Australia’s Physical Activity and Sedentary Behaviour Guidelines. Fact Sheet: Young People (13–17 Years).

[30] Goodman A., Goodman R. (2009). Strengths and difficulties questionnaire as a dimensional measure of child mental health. J. Am. Acad. Child Adolesc. Psychiatry.

[31] Mellor D. (2005). Normative data for the Strengths and Difficulties Questionnaire in Australia. Aust. Psychol..

[32] Australian Bureau of Statistics Socio-Economic Indexes for Areas. http://www.abs.gov.au/websitedbs/censushome.nsf/home/seifa.

[33] R Core Team (2017). R: A Language and Environment for Statistical Computing.

[34] Costigan S.A., Barnett L., Plotnikoff R.C., Lubans D.R. (2013). The health indicators associated with screen-based sedentary behavior among adolescent girls: A systematic review. J. Adolesc. Health.

[35] Hoare E., Skouteris H., Fuller-Tyszkiewicz M., Millar L., Allender S. (2014). Associations between obesogenic risk factors and depression among adolescents: A systematic review. Obes. Rev..

[36] Tremblay M.S., LeBlanc A.G., Kho M.E., Saunders T.J., Larouche R., Colley R.C., Goldfield G., Gorber S.C. (2011). Systematic review of sedentary behaviour and health indicators in school-aged children and youth. Int. J. Behav. Nutr. Phys. Act..

[37] Pagani L.S., Fitzpatrick C., Barnett T.A., Dubow E. (2010). Prospective associations between early childhood television exposure and academic, psychosocial, and physical well-being by middle childhood. Arch. Pediatr. Adolesc. Med..

[38] Gunnell K.E., Flament M.F., Buchholz A., Henderson K.A., Obeid N., Schubert N., Goldfield G.S. (2016). Examining the bidirectional relationship between physical activity, screen time, and symptoms of anxiety and depression over time during adolescence. Prev. Med..

[39] Lissak G. (2018). Adverse physiological and psychological effects of screen time on children and adolescents: Literature review and case study. Environ. Res..

[40] Álvarez-Bueno C., Pesce C., Cavero-Redondo I., Sánchez-López M., Martínez-Hortelano J.A., Martínez-Vizcaíno V. (2017). The effect of physical activity interventions on children’s cognition and metacognition: A systematic review and meta-analysis. J. Am. Acad. Child Adolesc. Psychiatry.

[41] White R.L., Babic M.J., Parker P.D., Lubans D.R., Astell-Burt T., Lonsdale C. (2017). Domain-specific physical activity and mental health: A meta-analysis. Am. J. Prev. Med..

[42] Kulkarni A., Swinburn B., Utter J. (2015). Associations between diet quality and mental health in socially disadvantaged New Zealand adolescents. Eur. J. Clin. Nutr..

[43] Renzaho A., Kumanyika S., Tucker K.L. (2011). Family functioning, parental psychological distress, child behavioural problems, socio-economic disadvantage and fruit and vegetable consumption among 4–12 year-old Victorians, Australia. Health Promot. Int..

[44] Mihrshahi S., Myton R., Partridge S.R., Esdaile E., Hardy L.L., Gale J. (2019). Sustained low consumption of fruit and vegetables in Australian children: Findings from the Australian National Health Surveys. Health Promot. J. Aust..

[45] Halfon N., Larson K., Slusser W. (2013). Associations between obesity and comorbid mental health, developmental, and physical health conditions in a nationally representative sample of US children aged 10 to 17. Acad. Pediatr..

[46] Twenge J.M., Joiner T.E., Rogers M.L., Martin G.N. (2018). Increases in depressive symptoms, suicide-related outcomes, and suicide rates among US adolescents after 2010 and links to increased new media screen time. Clin. Psychol. Sci..

