# Supplementary material for: Healthy Lifestyle Behaviours Are Associated with Children’s Psychological Health: A Cross-Sectional Study
Source: Int J Environ Res Public Health. 2020 Oct 15;17(20):7509. doi: 10.3390/ijerph17207509 (PMC7602583; doi:10.3390/ijerph17207509)
Supplement: Supplementary file 1 [file ijerph-17-07509-s001.pdf]

Supplementary Material

Table 1. Healthy lifestyle behaviour cut-offs used in the analysis (half serves in the recommendations were rounded up to whole serves).

Indicator	Healthy (met indicator)	Unhealthy (did not meet indicator)
Fruit recommendations (serves per day)		
5-8 years	≥2[a]	<2
9-15 years	≥2	<2
Vegetables recommendations (serves per day)		
5-8 years	≥5[b]	<5
9-11 years	≥5	<5
12-15 years, girls	≥5	<5
12-15 years, boys	≥6[c]	<6
Discretionary foods (serves per week)		
5-15 years	≤3	>3
Average time in PA/day (minutes)		
5-15 years	≥60	<60
Average time screen time/day (hours)		
5-15 years	<2	≥2

[a] 1.5 in National Health and Medical Research Committee (NHMRC) Dietary Guidelines (27).

[b] 4.5 in National Health and Medical Research Committee (NHMRC) Dietary Guidelines (27).

[c] 5.5 in National Health and Medical Research Committee (NHMRC) Dietary Guidelines (27).

Table 2. Unadjusted associations between diet/physical activity indicators and scoring in the at-risk range for each of the Strength and Difficulty Questionnaire (SDQ) subscales.

	**Emotional Symptoms OR (95% CI)**		**Conduct Problems OR (95% CI)**		**Hyper-activity OR (95% CI)**		**Peer Problems OR (95% CI)**		**Prosocial Behaviour OR (95% CI)**	
**Indicator**	**5-10 years**	**11-15years**	**5-10years**	**11-15years**	**5-10years**	**11-15years**	**5-10years**	**11-15years**	**5-10years**	**11-15years**
Met fruit recommendation	0.70 (0.42,1.17)	0.73 (0.45-1.19)	0.83 (0.45,1.54)	0.67 (0.33-1.33)	0.70 (0.43-1.14)	1.19 (0.67-2.09)	0.49 (0.26-0.92)*	0.75 (0.44-1.26)	0.83 (0.27-2.56)	1.16 (0.44-3.08)
Met vegetable recommendation	0.36 (0.07,1.91)	0.67 (0.23-2.01)	0.57 (0.14,2.38)	0.41 (0.12-1.46)	0.46 (0.17-1.27)	0.45 (0.16-1.33)	0.97 (0.29-3.25)	1.04 (0.38-2.82)	NA#	0.34 (0.6-1.92)
Met discretionary food limit	0.82 (0.49,1.36)	0.78 (0.48-1.27)	0.44 (0.22,0.88)*	0.53 (0.27-1.04)	0.61 (0.37-1.00)	0.64 (0.37-1.12)	0.71 (0.37-1.34)	0.73 (0.44-1.23)	0.06 (0.02-0.18)*	0.62 (0.21-1.83)
Met PA recommendation	0.60 (0.36-1.02)	0.74 (0.43-1.27)	0.67 (0.36-1.26)	0.89 (0.44-1.82)	1.20 (0.72-1.99)	0.92 (0.52-1.63)	0.61 (0.32-1.16)	0.37 (0.20-0.70)*	0.74 (0.24-2.27)	0.95 (0.33-2.72)
Met screen time recommendation	0.46 (0.27-0.78)*	0.61 (0.36-1.04)	0.42 (0.22-0.80)*	0.70 (0.33-1.48)	0.44 (0.26-0.72)*	0.53 (0.29-0.96)*	0.35 (0.18-0.69)*	0.68 (0.38-1.20)	0.18 (0.06-0.56)*	0.25 (0.06-0.97)*

*p<0.05, #Insufficient responses to report this measure.

International Journal of
Environmental Research and Public Health

MDPI

Table 3. Demographics included in multivariable analysis of diet and physical activity factors predictive of total difficulties score in children aged 5-15 years, stratified by age.

**Mean Score Difference**	**5-10 years**	**11-15 years**
Sex (ref: female)	-1.59 (-2.65, -0.55)**	-1.26 (-2.41, -0.10)*
Mother completed high school/diploma (ref: University degree)	0.36 (-1.34, 2.05)	3.45 (1.80, 5.09) ***
Mother completed year 10 or below (ref: University degree)	1.26 (0.16, 2.35)**	1.03 (0.01, 2.06)*
Age	-0.01 (-0.26, 0.25)	-0.21 (-0.56, 0.15)
Single parent family	1.93 (0.71, 3.14)*	2.08 (0.69, 3.46)**
Living regional or remote (ref: Major cities)	-1.27 (-2.22, -0.32)**	0.63 (-0.55, 1.80)
BMI: overweight/obese	0.54 (-0.61, 1.70)	1.15 (-0.57, 2.87)

*** = P<0.001, **= P <0.01, *=P<0.05. All factors were included in a single multivariable model, adjusted for age, sex, BMI, mother's highest qualification, family structure (single parent family), and remoteness.
